# Numerical Simulation of Thrombotic Occlusion in Tortuous Arterioles

**Published:** 2017-12-06

**Authors:** Zhi-Gang Feng, Miguel Cortina, Jennifer KW Chesnutt, Hai-Chao Han

**Affiliations:** 1 Department of Mechanical Engineering, USA; 2 Biomedical Engineering Program, UTSA-UTHSCSA, USA

**Keywords:** Thrombosis, Lattice-Boltzmann method, Shear stress, Flow, Microvessel tortuosity, Platelet activation, Platelet size effect

## Abstract

Tortuous microvessels alter blood flow and stimulate thrombosis but the physical mechanisms are poorly understood. Both tortuous microvessels and abnormally large platelets are seen in diabetic patients. Thus, the objective of this study was to determine the physical effects of arteriole tortuosity and platelet size on the microscale processes of thrombotic occlusion in microvessels. A new lattice-Boltzmann method-based discrete element model was developed to simulate the fluid flow field with fluid-platelet coupling, platelet interactions, thrombus formation, and thrombotic occlusion in tortuous arterioles. Our results show that vessel tortuosity creates high shear stress zones that activate platelets and stimulate thrombus formation. The growth rate depends on the level of tortuosity and the pressure and flow boundary conditions. Once thrombi began to form, platelet collisions with thrombi and subsequent activations were more important than tortuosity level. Thrombus growth narrowed the channel and reduced the flow rate. Larger platelet size leads to quicker decrease of flow rate due to larger thrombi that occluded the arteriole. This study elucidated the important roles that tortuosity and platelet size play in thrombus formation and occlusion in arterioles.

## Introduction

Blood vessel tortuosity is a common occurrence in humans [1]. Tortuous microvessels are found throughout the human body, including the heart [2], brain [3,4], and eye [5,6]. Compared with a straight path, microvessel tortuosity changes blood flow to alter blood cell trajectories and fluid shear stress. For example, *in vivo* and *in silico* studies demonstrated that tortuosity increased shear stress to activate platelets and initiate thrombus formation [7–9]. As a thrombus grows, the flow of blood may be reduced or blocked, which can result in myocardial infarction or stroke that may lead to death. Microvascular thrombi have been observed in clinical, experimental, and autopsy situations [10,11]. As well, microvascular tortuosity and thrombosis are both observed clinically in diabetes mellitus, and diabetic patients have a high risk of death due to thrombosis [12,13]. Because of these observations and the fact that thrombosis is a major contributor to cardiovascular disease, which is the leading cause of death worldwide, the study of thrombus formation and thrombotic occlusion in tortuous microvessels is of clinical importance.

Platelets are the main cellular components of microvascular thrombi [14]. Platelets become activated under thrombogenic conditions in order to adhere to the endothelial wall and aggregate with each other. Adhesion and aggregation occur through glycoproteins that are embedded in the platelet surface membrane, various plasma proteins, and networks of fibrin [15]. High shear stresses, along with chemical agonists, induce platelet activation. Activated platelets activate additional platelets by releasing and catalyzing platelet agonists [15]. Once thrombi begin to form, shear stresses within the vessel may be increased to also activate additional platelets, possibly resulting in occlusion. Thrombotic occlusion has been visualized *in vitro* in stenoses [16–18] and *in vivo* in tortuous venules [9]. However, the microscale processes involved are difficult to observe in such studies. On the other hand, computational models have elucidated some microscale processes in thrombus formation through simulation of individual platelets that were either activated due to chemical agonists or endothelial injury [19–27], activated due to high shear stress [7,8], or able to aggregate without activation [28]. Most of these previous computational studies examined straight vessels or channels, though some included geometric features such as stenoses [22,27], baffles [28], or tortuosity [7,8]. Polanczyk et al. studied the thrombus formation by comparing the blood shear thinning rate of each computational cell with a pre-defined critical value [29]. A cell is treated as a thrombus if its shear thinning rate is equal to or less than the critical value. Govindarajan et al. recently considered thrombus as porous media and used a computational model to study its growth [30]. Zimny et al. proposed a multi-scale approach to simulate blood flow and thrombus formation in intracranial aneurysms [31]. However, most of these studies did not examine activation of platelets by shear stress, and none investigated thrombotic occlusion.

In addition to high shear stress, platelet size is also a factor present in conditions associated with thrombotic complications, as well as bleeding complications. For normal healthy human subjects, an increase in platelet size yielded an increase in platelet aggregation in platelet rich plasma [32]. Larger platelet size is observed in pathological conditions, including diabetes mellitus [33], hypertrophic cardiomyopathy [34], acute myocardial infarction [35], restenosis following coronary angioplasty [35], pulmonary hypertension [36], and giant platelet disorders [37]. Smaller platelet size is observed in other pathological conditions, such as reactive systemic amyloid A amyloidosis [38] and Wiskott-Aldrich syndrome [39]. Hence, platelet size could be an important factor in thrombus formation in various pathologies.

The lattice-Boltzmann method (LBM) has been extensively used in blood flow simulations with or without red blood cells and platelets. Ouared and Chopard studied blood flow, non-Newtonian rheology, and clotting processes with the LBM [40]. Zhang et al. applied the LBM to study red blood cell aggregation and dissociation in shear flows [41]. As well, Sun et al. used the LBM to investigate the effect of red blood cells on leukocyte rolling [42]. The state-of-the-art on the use of LBM in modeling complex flow can be found in a recent review paper [43]. The LBM has been shown to provide an excellent platform to simulate cells in blood flow.

Accordingly, the objective of this study was to determine the physical effects of vessel tortuosity and platelet size on the microscale processes of thrombotic occlusion in microvessels. In this study, a new lattice-Boltzmann method based discrete element model (DEM), i.e. LBM-DEM, has been developed to simulate the fluid-particle interactions, platelet-platelet interactions, and thrombus formation. The thrombus initiation, growth and possible occlusion in arterioles were simulated. We also investigated the effect of platelet size, which is often larger in diabetic patients, on thrombus formation.

### Numerical simulation methods

The study of transport, collision, activation, and adhesion of platelets involved in thrombus formation and the effect of thrombus development on the flow fields in tortuous venules and arterioles requires solving the fluid fields and platelet dynamics simultaneously. In this section we will briefly describe both the numerical method used for solving the flow field and the discrete element model used for modeling platelet activation, transport, and adhesion. To simplify the computation, fluid flow is assumed to be two-dimensional (2D) and platelets are modeled as spherical particles whose movements are restricted within the plane [8]. Red blood cells and white blood cells are neglected throughout the simulations.

### LBM simulation of fluid velocity

The fluid fields are simulated by lattice Boltzmann method, in which fluid motion is modeled by the transport of fluid particles on the lattice sites of a uniform Cartesian grid. These fluid particles move along a limited number of specified directions from one lattice site to a neighboring site. They move at a discrete velocity per time-step in two steps: they first stream to neighboring sites, and then they collide with fluid particles there. A distribution function, *f_i_*(*x⃗*,*t*), is used for representing the populations of particles residing at node *x⃗* at time *t* and moving in the *i*-th direction. The evolution of the fluid particle distributions resulting from the collision and streaming processes are modeled using the single-relaxation BGK *(Bhatnagar-Gross-Krook)* model [44],

(1) $$f_{i}\left(\vec{x}+{\vec{c}}_{i}\delta t,t+\delta t\right)=f_{i}\left(\vec{x},t\right)-\frac{1}{\tau }\left[f_{i}\left(\vec{x},t\right)-f_{i}^{\left(eq\right)}\left(\vec{x},t\right)\right]$$

where *c⃗_i_* is the fluid particle velocity along the *i*-th direction, $f_{i}^{\left(eq\right)}$ is the distribution function at equilibrium, and *τ* is the relaxation time. The fluid density *ρ* and velocity field *u⃗* are obtained from *f_i_*(*x⃗,t*) as follows:

(2) $$\rho \left(\vec{x},t\right)=\sum_{i}f_{i}\left(\vec{x},t\right)$$

and

(3) $$\rho \left(\vec{x},t\right)\vec{u}\left(\vec{x},t\right)=\sum_{i}f_{i}\left(\vec{x},t\right){\vec{c}}_{i}$$

The equilibrium distribution function, $f_{i}^{\left(eq\right)}$, can be determined by the local velocity *u⃗* using the following equation:

(4) $$f_{i}^{\left(eq\right)}\left(\vec{x},t\right)=\rho w_{i}\left[1+3\left({\vec{c}}_{i}\cdot \vec{u}\right)+\frac{9}{2}{\left({\vec{c}}_{i}\cdot \vec{u}\right)}^{2}+\frac{3}{2}\left(\vec{u}\cdot \vec{u}\right)\right]$$

where *w_i_* are the values of the weights. For a D2Q9 LBM model [45], these weights are chosen to be:

(5) $$w_{0}=\frac{4}{9},\;\;\;w_{1}=w_{3}=w_{5}=w_{7}=\frac{1}{9},\;\;\;w_{2}=w_{4}=w_{6}=w_{8}=\frac{1}{36}$$

The relaxation time, *τ*, is related to the kinematic viscosity of the fluid, *v*, by the following equation,

(6) $$\tau =\frac{1}{2}\left(6\nu +1\right)$$

To simulate the interactions between platelets and fluid, an unresolved discrete element model is used to keep computational cost manageable [46]. The coupling between platelets and fluid is achieved by adding the fluid-platelet interaction force or drag force into the collision step of LBM [47]:

(7) $$f_{i}\left(\vec{x}+{\vec{c}}_{i}\delta t,t+\delta t\right)=f_{i}\left(\vec{x},t\right)-\frac{1}{\tau }\left[f_{i}\left(\vec{x},t\right)-f_{i}^{\left(eq\right)}\left(\vec{x},t\right)\right]+\frac{3}{2}w_{i}{\vec{F}}_{b}\cdot {\vec{c}}_{i}$$

Tortuous segments of vessels are modeled as 2D channels in the shape of cosine curves of two periods. The diameter of the channels is D. The centerline curve of the channel is a cosine curve defined by

(8) $$g\left(x\right)=A\cos\left(\frac{2\pi x}{\lambda }\right),\;\;\;0\leq x\leq 2\lambda$$

where A is amplitude and λ is wavelength. Following the study by Chesnutt and Han [8], we introduce a tortuosity index TI as the ratio of amplitude to the wavelength of the centerline curve, that is,

(9) $$T=\frac{A}{\lambda }$$

In LBM simulation, a lattice domain with uniform grid spacing is used to cover a rectangular region of 0 ≤ *x* ≤ 2*λ* and 0 ≤ *y* ≤ 2*A* + *D.* Two types of lattice nodes, fluid nodes and solid nodes, are defined based on the tortuosity of the channel. Flow is driven by a net positive pressure at the inlet. Periodic boundary conditions are applied at the inlet and outlet. A detailed implementation of LBM can be found in an earlier study by Feng and Michaelides [47].

These equations for fluid flow are combined with equations for the motion of platelets described below.

### Discrete element method of platelet dynamics

To model the transport, collision, aggregation, and adhesion of platelets in tortuous venules and arterioles, the forces and torques acting on the platelets are computed at every simulation time step. In the remainder of this subsection, the term *adhesion* refers to both adhesion of platelets to the wall that form thrombi and to aggregation of platelets with each other, which may occur in the bulk flow. The following equations of motion are solved to obtain platelet translational and rotational velocities,

(10) $$m_{p}\frac{d\vec{v}}{dt}={\vec{F}}_{d}+{\vec{F}}_{\mathrm{coll}}+{\vec{F}}_{\mathrm{adh}}$$

and

(11) $$\frac{d\Omega_{z}}{dt}=M_{d,z}+M_{\mathrm{coll},z}$$

Here, drag force *F⃗_d_*, platelet-platelet or platelet-wall collision force *F⃗_coll_*, and adhesion force *F⃗_adh_* are the forces considered that act over a platelet. The buoyancy force and gravity force are neglected because the density of a platelet is very close to the density of fluid. The torques acting over a platelet include fluid induced torque *M_d_*_,_*_z_* and collision induced torque *M_coll,z_*. Note that as per equation (10), two platelets that are adhering will detach if drag and collision forces overcome the adhesion force. The movements of platelets are primarily caused by fluid motion; platelets move along the blood stream with low relative velocity, resulting in very low particle Reynolds numbers (in the order of 0.01 for most cases). Thus, the drag force acting on platelets can be computed accurately using the Stokes law:

(12) $${\vec{F}}_{d}=-6\pi a\mu \left({\vec{V}}_{p}-{\vec{V}}_{f}\right)$$

where *a* is the platelet radius, *μ* is the fluid dynamic viscosity, and (*V⃗_p_ – V⃗_f_*) is the relative velocity of the platelet. The fluid viscosity is a constant property of fluid; it has a significant effect on the drag force of platelets in flows of low Reynolds numbers. The fluid velocity and platelet velocity are updated at each simulation time step.

The collision force is computed when platelets hit each other or the wall. This force is modeled using a soft sphere collision model [47], in which the normal and tangential components of the collision force are described as a combination of elastic and damping forces:

(13) $${\vec{F}}_{\mathrm{coll}}=-k_{\mathrm{coll}}\vec{\delta }+\eta_{\mathrm{coll}}{\vec{v}}_{\mathrm{rel}}$$

where *δ⃗* is the overlap displacement of the platelets, *k_coll_* is the elastic coefficient, *v⃗_rel_* is the relative velocity of the platelets, and *η_coll_* is the damping coefficient. The elastic and damping coefficients used are: *k_coll_* = 0.005 N/m and *η_coll_ =* 0.005 N/m. The selection of these parameters is to ensure that the collision force is able to prevent significant overlapping between platelets.

There are a few platelet adhesion models existing in the literature. For example, Wu et al. [48], recently introduced a 3D platelet-vessel wall adhesion model in which they modeled platelet-wall adhesion at the single receptor level. In our model, platelet adhesion is modeled by a spring model proposed by Kamada et al. [21]. The adhesion force is computed only if activated platelets collide with other platelets or the wall. The direction of this force is the normal direction to the point of contact. This force was computed using the following equation.

(14) $${\vec{F}}_{\mathrm{adh}}=k_{\mathrm{adh}}\delta_{n}\vec{n}$$

In the present study the elastic coefficient used to compute this force is *k_adh_* =0.008 N/m. In addition to providing computational efficiency, this elastic coefficient was chosen because it yields an adhesion force on the order of 10^2^ pN, assuming a displacement *δ_n_* on the order of 10 nm. This adhesion force value falls within the range of *in vitro* estimates that are on the order of 10^0^ to 10^4^ pN [49–51], and the value is within one order of magnitude of that (32 pN) used by another computational model related to thrombectomy [52].

The fluid moment on a platelet due to local fluid vorticity is calculated as [8],

(15) $$M_{F,z}=8\pi \mu a^{3}\;\left(\omega_{xy}-\Omega_{z}\right)$$

where *ω_xy_* is the local fluid vorticity. For simplicity, the period of hesitation due to resistance of rolling of one platelet over another is neglected in the simulations.

The activation and aggregation of platelets, i.e., the formulation of thrombus, is simulated using the shear-induced activation model developed by Chesnutt and Han, in which a platelet becomes activated if it experiences a shear stress above a critical shear stress [8]. With this assumption, physiological shear stress does not activate a platelet, irrespective of the amount of time a platelet is subjected to the physiological shear stress. A justification for neglecting time dependency of activation is given in the Discussion and Conclusions section. To account for the presence of chemical agonists released by activated platelets, we assume a platelet becomes activated if it contacts another activated platelet. Furthermore, only activated platelets are subject to adhesion with each other and the endothelium to form a thrombus, and activation is considered irreversible. To make the computational cost feasible, the lower platelet adhesive strength at initial attachment is neglected, and the higher adhesive strength after platelet spreading and stabilization is modeled by the chosen elastic coefficient in equation (14).

### Simulation parameters and conditions

To investigate the effect of vessel tortuosity, three tortuosity levels (TI = 0.08, 0.16 and 0.24) were simulated. The amplitude A and wavelength *λ* were varied to obtain vessels with these TI. However, all cases have the same vessel arc length. Most simulations used a platelet size of 2.4 μm unless specified otherwise. A diameter of 2.4 μm is the average value for normal human platelets [33]. To study the effect of platelet size on the microscale processes of thrombotic occlusion in microvessels, we chose three platelet sizes of diameter 1.9 μm, 2.4 μm, and 3.1 μm for a vessel with medium tortuosity of TI = 0.16 at a constant pressure drop [7].

The vessel curvature could have an impact on fluid dynamics in 3D and Dean number can be used to assess curvature effects. The Dean number is defined as:

(16) $$Dn=\frac{\rho VD}{\mu }\sqrt{\frac{D}{R_{C}}}$$

Here, *R_c_* is the radius of curvature. In the case of A=D=25 μm and TI=0.24, the maximum radius of curvature for the cosine curve defined in Eqn. 8 is found to be $R_{c}=\frac{1}{A{\left(\frac{2\pi }{\lambda }\right)}^{2}}\approx 11\;\mu m$. For all the cases considered in the present study, it is found that the Dean number is in the order of 0.1, such that secondary flow is negligible. Therefore, the lack of secondary flow associated with vessel curvature in our 2D simulations would be similar to that in 3D.

In human and animal circulations, small alterations such as stenosis or tortuosity in a vessel branch will not have much effect on pressure and flow in the vessel due to the physiological compensation mechanisms. However, severe stenosis and tortuosity may significantly reduce the flow. Since the microvasculature is often composed of vessel networks with collateral flow, it is reasonable to assume that the pressure drop in a given arteriole remains constant during the narrowing of vessel lumen. In computational simulations, the effects of stenosis and aneurysm etc. are often simulated at either a constant flow rate or constant pressure drop [7,8,53]. Therefore, we considered these two types of flow conditions for the thrombosis simulation in this study.

First, we simulated the thrombus formation and growth process by assuming that the initial flow rate was the same for three different levels of TI. The pressure drops were determined accordingly and were then assumed to remain unchanged during the thrombus formation and growth process. Secondly, we simulated the thrombus formation and growth by assuming that the pressure drop remains constant for three different levels of TI.

## Simulation Results

For all simulations, the diameter of the vessel is D=25 μm. The length of the channel is L=314 μm. The properties of the fluid are selected to be similar to plasma properties: the fluid density *ρ* is 1030 kg/m^3^ and the kinematic viscosity *v* is chosen to be 1.2×10^−6^ m^2^/s.

### Flows in a straight channel: model validation

In order to validate the lattice Boltzmann method used for solving fluid velocity field, we simulate the flow rate and the shear stress for a plane Poiseuille flow in a straight channel and compare the simulation results with the analytical solutions. The LBM simulation uses 600 lattices in the direction of the channel and 48 lattices in the direction perpendicular to the channel. This results in a uniform grid spacing of Δ*_x_* = 0.523 μm. A constant time step of Δ*_t_* = 2×10^−7^ s is used for the simulation. A constant pressure drop between the inlet and outlet is prescribed; high pressure drop induces high fluid velocity. We have tested five cases with the pressure drops Δ*p* being 5 Pa, 10 Pa, 15 Pa, 20 Pa, and 30 Pa. No-slip boundary conditions are assumed at the wall of the channel.

The flow rate Q and the shear stress at the wall *τ_w_* are computed at steady state and they are compared with the following exact solutions of plane Poiseuille flows:

(17) $$Q=\frac{H^{3}}{12\mu }\frac{\Delta p}{L}$$

and

(18) $$\tau_{w}=\frac{H}{2}\frac{\Delta p}{L}$$

Both the simulation results and analytical results are listed in table 1. It is seen that the LBM simulation results agree very well with the exact solutions for all of the five cases studied here. The difference between the simulation value and exact solution is less than 4%. This indicates that the LBM with the chosen simulation parameters can produce accurate results. We also tested the use of a coarse grid of 300 × 24 and found the differences between the LBM and exact solutions to be generally within 8%.

### Simulation of platelets in a tortuous vessel

The motion and aggregation of platelets were simulated in channels of different TI under different boundary conditions to determine the effects of vessel tortuosity and platelet size on the formation and growth of thrombus and possible occlusion in tortuous arterioles. To lower the simulation cost, we used 320 lattices in the channel direction. Initially, 50 platelets are placed randomly in the channel. New platelets enter the channel randomly from the inlet at a rate that gave a time-averaged physiological concentration of 3 × 10^5^ platelets/mm^3^ in the absence of adhesion. Based on the normal shear stress in the straight microvessel, the critical shear stress for platelet activation is chosen to be 0.65 Pa for all tortuous vessel simulations, which is corresponding to a scaling factor of 1.07 [8].

### Effect of the channel tortuosity

Thrombus formation was simulated in channels of three tortuosity levels (TI=0.08, 0.16, and 0.24). The diameter of platelets was chosen to be 2.4 μm, which is the average value for normal human platelets [33].

For the cases of given initial flow rate, higher pressure drops were applied for channels of higher TI to achieve the same given initial flow rate (Table 2). It is seen that an increase of TI from 0.16 to 0.25 requires an increase of 20% in pressure drop to yield the same initial flow rate.

**Figure 1:**
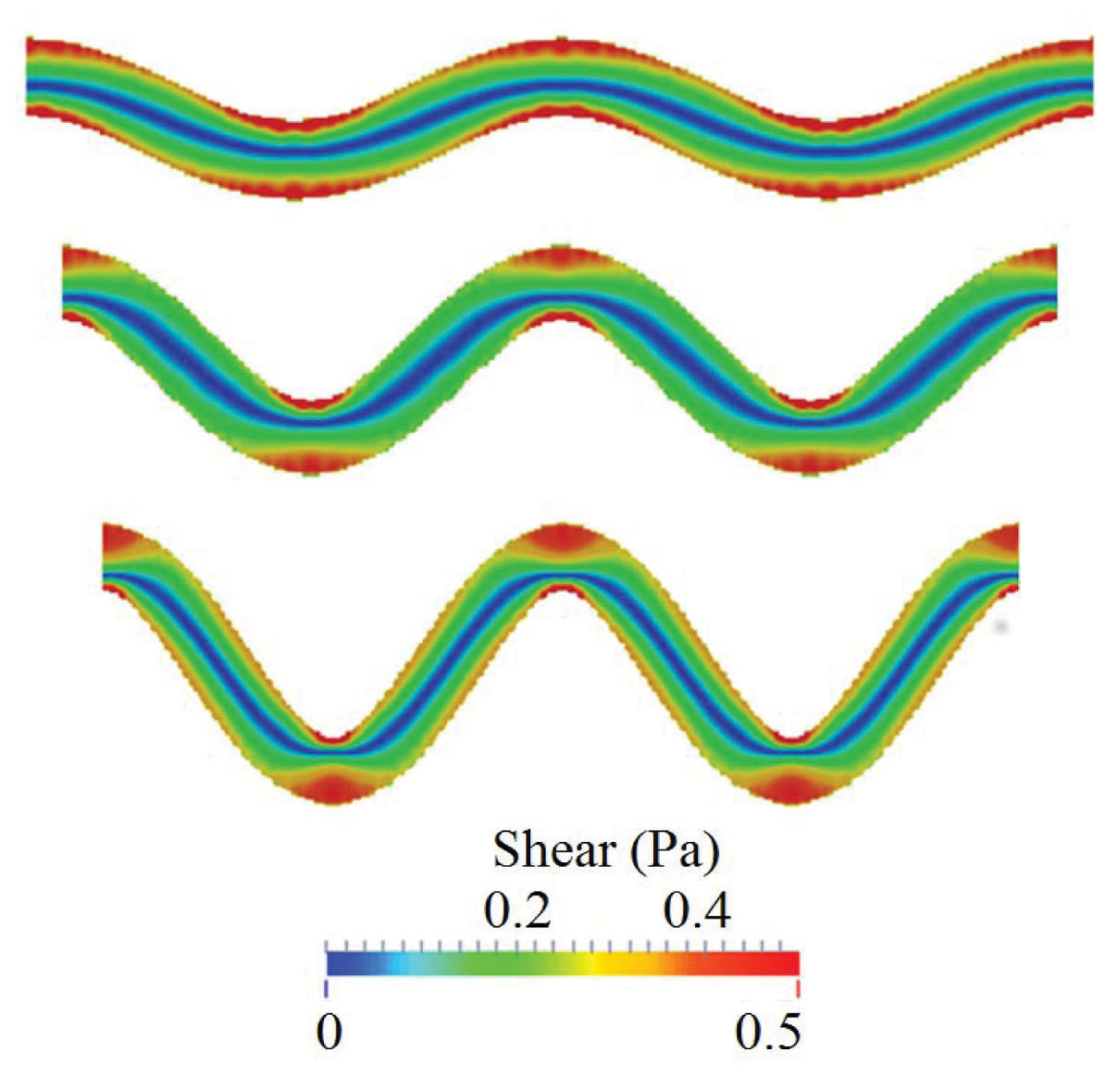
Initial shear stress distribution at the same flow rate in channels of three different tortuosity levels before platelet activations. From top to bottom, TI = 0.08, 0.16, and 0.24.

Under these conditions, the simulations showed that high shear stress zones occur near the vessel walls at the turning points, as shown in figure 1. Platelet activation and thrombus formation in a tortuous channel could initiate at these locations [8]. As tortuosity increases, the extent of the size of regions with high shear stress expands. That is, the critical shear stress region protrudes farther into the flow. The maximum shear stress in the channel also increases with the tortuosity index. It is also noted that the shear stress is higher at the curved bends of the wall, especially for the inner walls (inner side of the bend), for all the cases. The maximum shear stress values for TI = 0.16 and TI = 0.24 are within 10% difference of each other. It is also noted that the shear stress values for both of these cases are higher than the maximum shear stress for TI = 0.08 on the first and third bends of the channel. For the second bend, interestingly the value of shear stress for TI=0.08 is higher than the values obtained for TI = 0.16 and TI = 0.24. Note that the fluid shear stress constantly changes with time due to its interactions with platelets; this becomes more significant in regions near a platelet thrombus, where the formation of thrombus can change the surrounding flow fields significantly.

**Figure 2:**
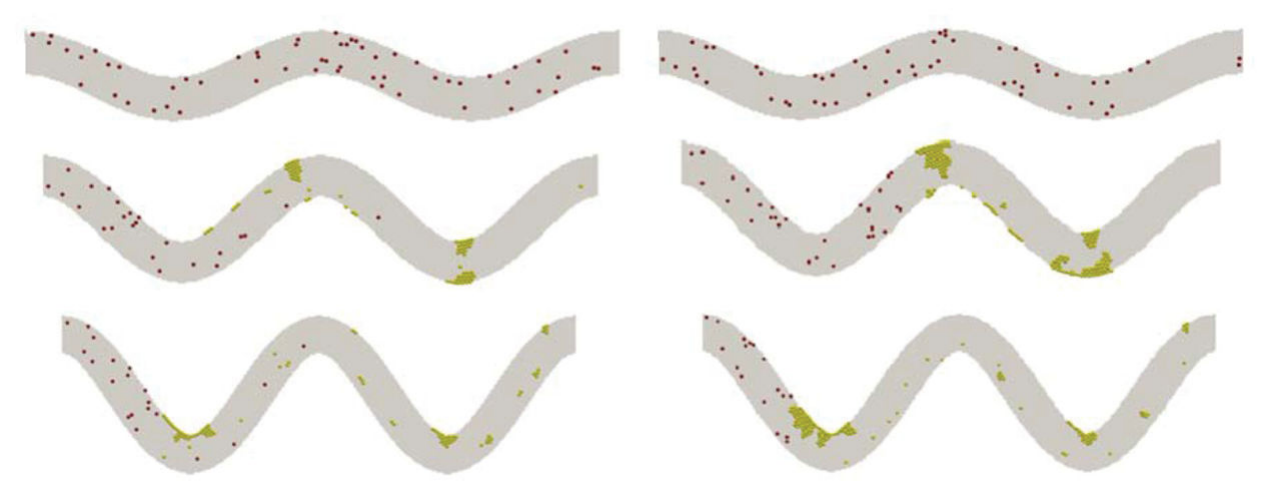
Growth of platelet thrombus over time for different tortuosity. From top to bottom: TI = 0.08, 0.16, and 0.24. Left: t = 0.5 s; right: t = 1 s.

Figure 2 shows snapshots of the aggregation of platelets in the channels of 3 levels of TI at t=0.5 s and t=1.0 s. For the case of TI=0.08, platelet activation does not occur during simulation as we expected because the maximum shear stress in the channel where platelets experienced a shear stress that is below the threshold value used for activation. Collisions between non-activated platelets are observed, but these colliding platelets do not form any thrombi. In the case where TI=0.16, there is a thrombus formation along the second and third curved bends of the channel. Both thrombi are increased in size by virtue of the activation and adhesion of the platelets that collide with them. In the case where TI=0.24, thrombus is formed similarly but at the first and third bends of the channel. It is seen that platelets are more likely adhered to the wall because of the higher shear stress near the wall. However, the collision of those initially non-activated platelets with the activated platelets or small thrombi contributes to the growth of thrombi, as seen in the cases of TI = 0.16 and TI = 0.24. This indicates that the channel tortuosity plays an important role in the activation and adhesion of platelets to the wall, but once the thrombus starts forming, platelet collisions become the dominant factor for platelet activation and thrombus growth.

**Figure 3:**
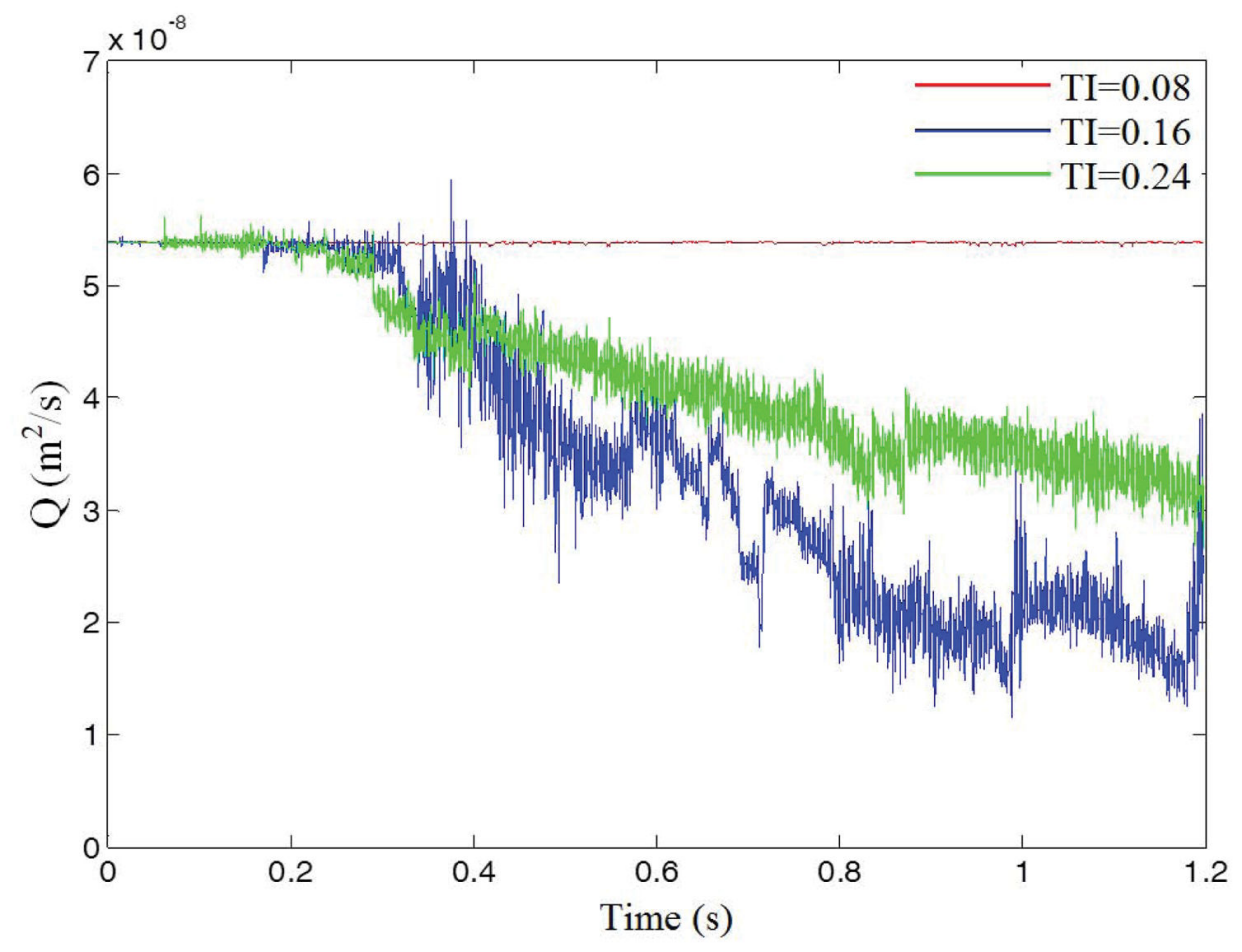
Flow rate over time for different tortuosity indexes.

The influence of the channel tortuosity on the flow rate is illustrated in figure 3. Before the initiation of thrombus, flow rates for these three cases are the same. As platelets occlude the vessel, the flow rate starts to decrease since the pressure drop over the vessel is kept constant. At time t=0.3s, the flow rates for TI=0.16 and TI=0.24 start to drop because of the formation of thrombus in the channels. After t=0.4s, the flow rate drops more drastically for TI=0.24 than for TI=0.16; this indicates that larger thrombi are blocking the channel and causing occlusion. Also, it is noticed that the formed thrombus is sometimes not stable and may be broken up by surrounding fluid as it grows, causing fluctuations of flow rate in the channels.

**Figure 4:**
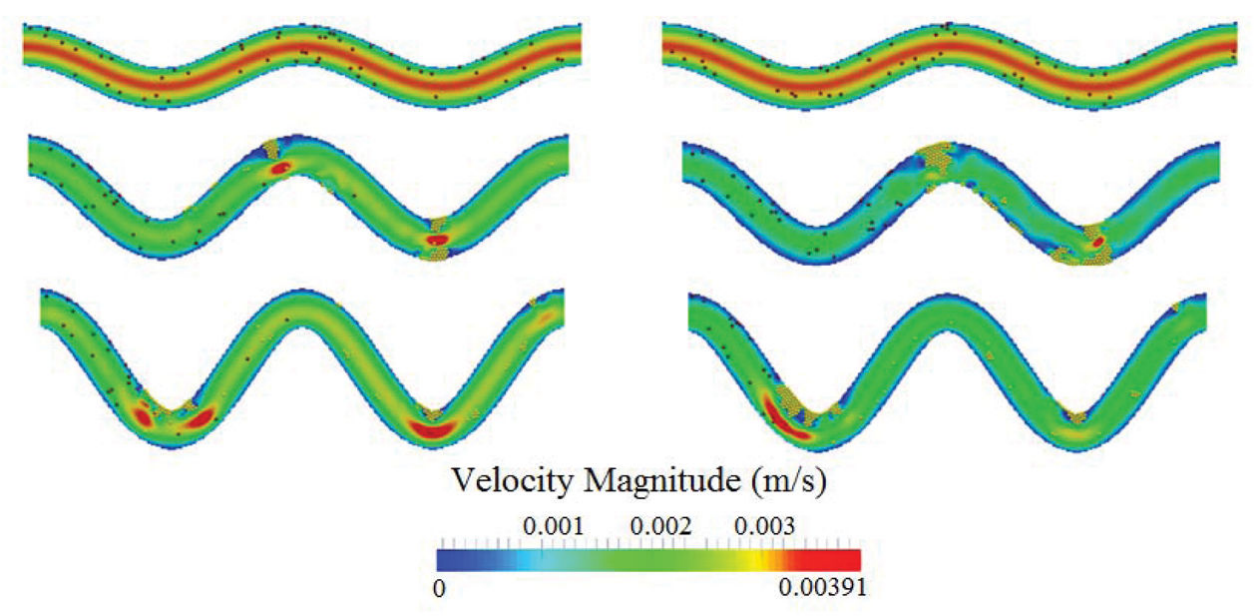
Comparison of flow velocity contour for three different tortuosity levels at two time points. Top to bottom: TI = 0.8, 0.16, and 0.24. Left: t = 0.5 s; right: t = 1 s.

The influence of the channel tortuosity on the flow field during the formation and growth of thrombus is illustrated in figure 4. It is seen that, as the thrombus grows, it narrows the channel. This decreases the overall flow rate in the channels. Though thrombus can also increase velocity and shear stress in the narrow section of the channel, the increase will activate and capture more platelets that eventually reduce the flow rate. Since the pressure gradient will not keep increasing to break up the thrombus, occlusion will occur and eventually block the channel.

**Figure 5:**
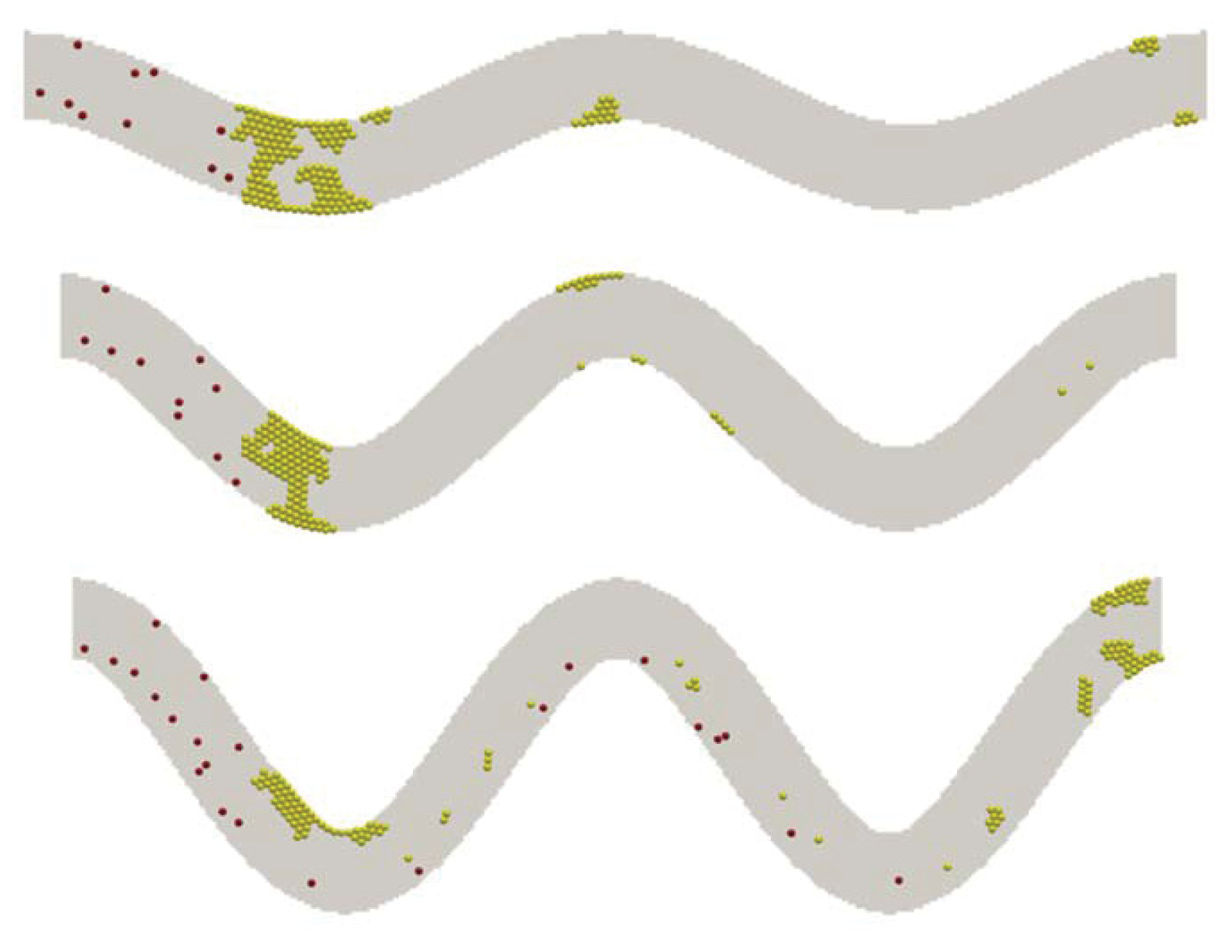
Platelet thrombus and occlusion at three levels of TI: 0.08 (top), 0.16 (middle), and 0.24 (bottom). Yellow spheres denote activated platelets and red spheres for non-activated platelets.

Simulations for tortuous microvessels under a given constant pressure drop of 20 Pa demonstrated similar patterns. Under these conditions, vessel tortuosity creates high shear stress zones near the wall around the turning points as well. The formations of the thrombus are depicted in figure 5 for the three levels of tortuosity. Occlusions are observed for TI=0.08 and 0.16, which completely block the flow in the channels.

**Figure 6:**
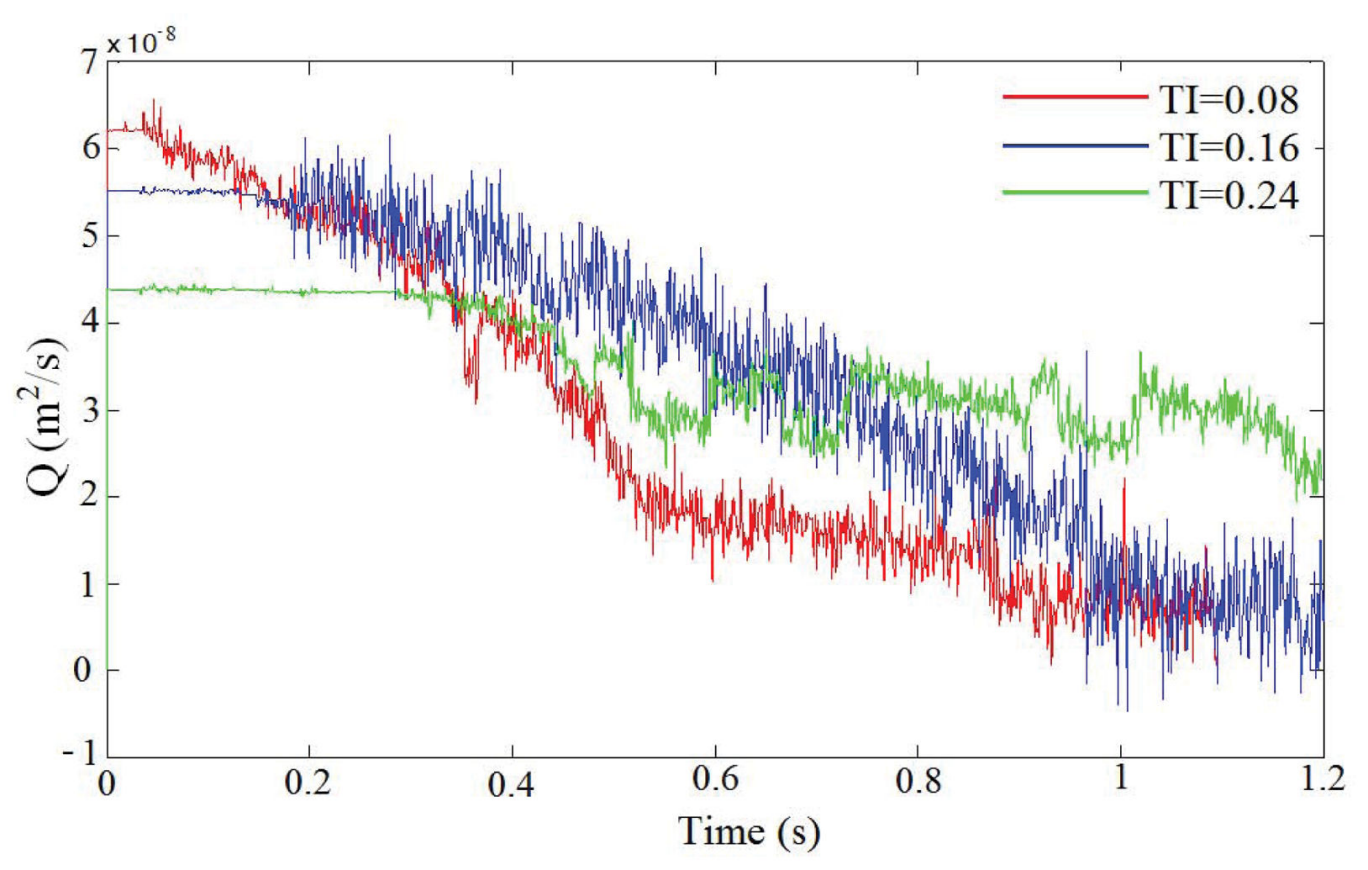
Flow rate plotted as a function of time for three TI values at a constant pressure drop of 20 Pa.

Figure 6 shows the flow rates obtained for the three tortuosity indexes. It is observed that initially the flow rate is higher for the cases with low tortuosity index under the same pressure drop. This higher flow rate leads to a higher wall shear stress and thus a high rate of platelet activation. It facilitates the formation and growth of thrombus. Thus, the flow rate in channels of low TI drops faster because of the formation of the thrombus, which blocks the flow. In the cases of TI=0.08 and 0.16, the growing thrombus eventually blocks the channels and causes occlusion.

### Effect of platelet diameter

**Figure 7:**
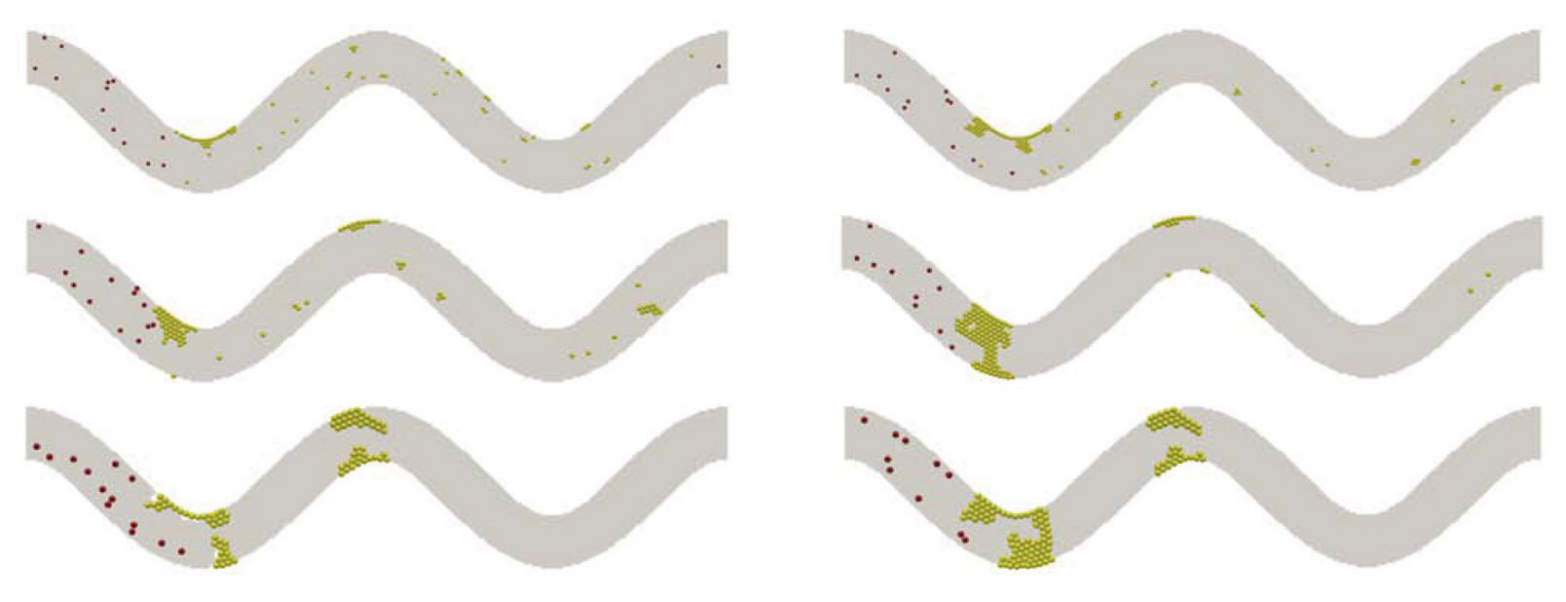
Agglomeration of platelets over time. From top to bottom: platelet diameter =1.9 μm, 2.4 μm, and 3.1 μm. Left: t=0.5 s; right: t=1.0 s.

**Figure 8:**
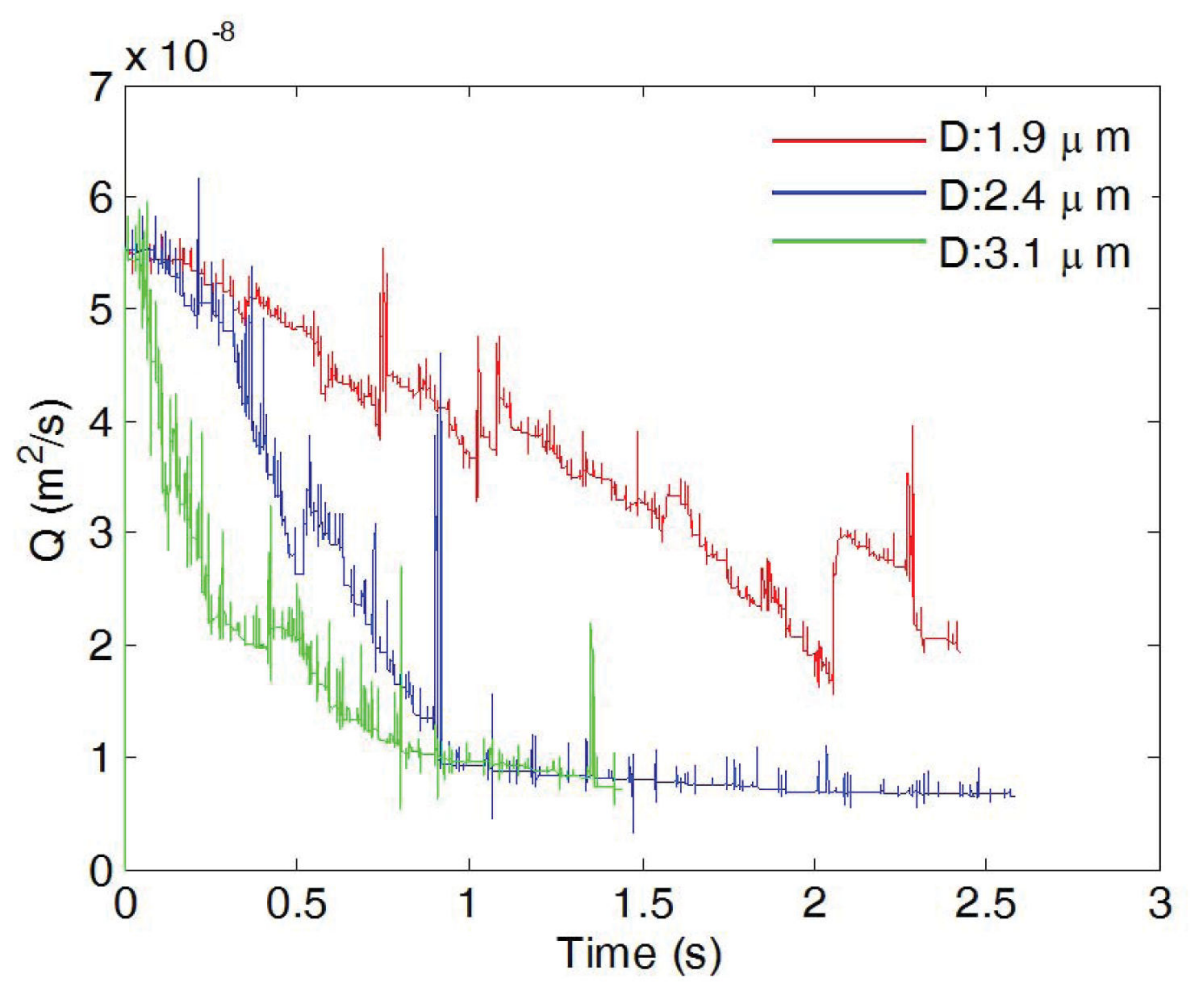
Flow rate plotted as a function of time during thrombus development for three platelet diameters.

**Figure 9:**
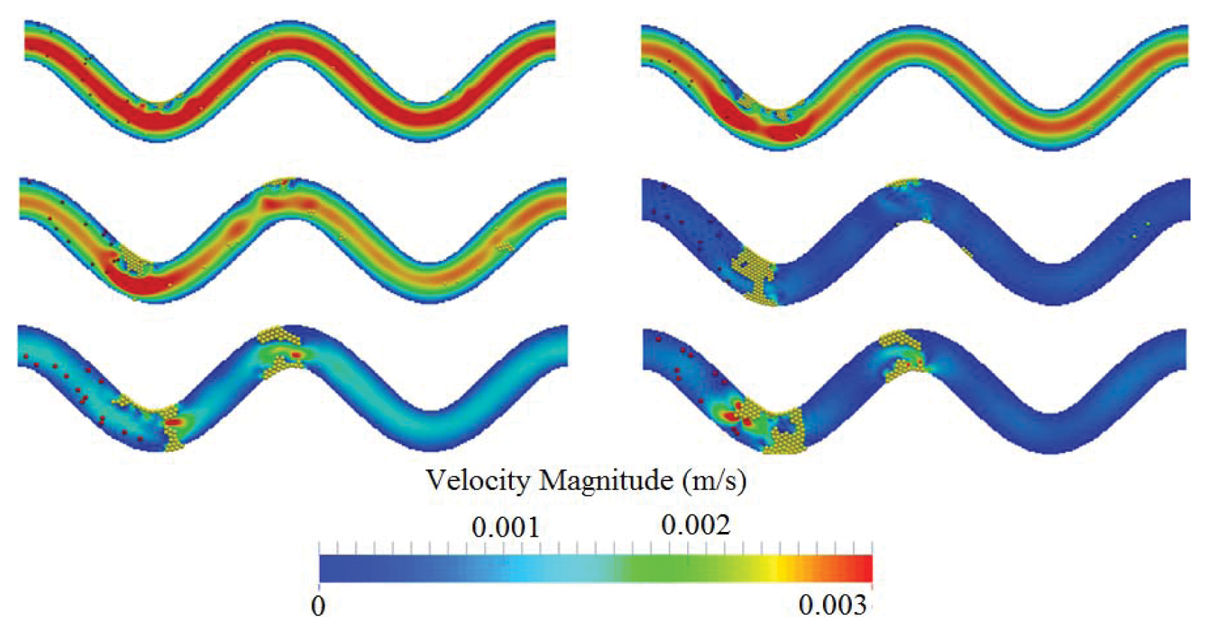
Comparison of flow velocity contour over time for three platelet sizes. From top to bottom: platelet diameter =1.9 μm, 2.4 μm, and 3.1 μm. Left: t = 0.5 s; right: t = 1.0 s. TI=0.16.

The platelet size affects the thrombus formation and growth as well. Figure 7 shows snapshots of platelets in a channel of TI=0.16 at t=0.5 s and t=1.0 s. The same critical shear stress for platelet activation (0.65 Pa) is chosen for each case. In all three cases studied here, the thrombus begins forming where the highest shear stress is, which is on the inner wall of the first bend of the channel. In the case of the platelet with a diameter of 1.9 μm, a small thrombus is formed on the inner wall of the first bend of the channel at t=0.5s, and more platelets start to adhere to this thrombus after colliding with it. However, for small platelets with diameter 1.9 μm, small thrombi that formed due to the collision between activated platelets flow downstream without adhering to the wall, and the channel flow rate is not considerably affected. In the case of normal platelets with diameter 2.4 μm, there are also thrombus formations on the inner wall of the first bend and the outer wall of the second bend. The formation of these thrombi occurred at an earlier time than the preceding cases. Also, the size of the thrombus and the number of activated platelets are significantly larger than the case in which platelets have a diameter 1.9 μm. Occlusion is observed for the case with normal platelets, and the channel was completely blocked by the thrombus that formed at the first bend. As the platelet diameter is further increased, the size and the number of activated platelets of the thrombus located at the first bend increase. This fact is evident after observing the results obtained in the case where platelet diameter is 3.1 μm. The thrombus formation for this case occurs earlier than the previous cases for normal platelets (diameter=2.4μm). In addition, more thrombi begin forming at the inner and outer walls of the second bend. As a result of an increment in the size of the platelet, the total blockage of the channels also occurs earlier than in the case of normal platelets. In all of the three cases, thrombus formation and posterior blockage of the channel modify the velocity profile and change flow rate over time. This effect is illustrated in figures 8,9 where flow rate is observed to diminish over time in all the cases. For the case of small sized platelets with a diameter of 1.9 μm, it takes longer to both form a significant thrombus and partially block the channel. Even then, the formed thrombus is less stable. The reduction of flow rate is more severe in the cases where platelet diameters are 2.4μm or 3.1μm, and the channel was completely blocked by the thrombus; for the case of diameter 1.9 μm, the flow rate decreases, but not as drastically as in the previous cases.

Figure 9 shows the channel velocity contours and actual thrombi for all three cases where platelet size varies at t=0.5 s and t=1.0 s. Clearly, the size of platelets plays a significant role in platelet occlusions. The increase of platelet size facilitates the formation of thrombi and increases the chance of occlusions.

## Discussion and Conclusions

We have developed a lattice Boltzmann method based approach to simulate the platelet activation and thrombus formation process in tortuous channels. The advantage of this approach is that it allows the coupling of fluid and platelets, giving a more realistic representation of platelet aggregation and thrombus formation. Our results demonstrated that arteriole tortuosity can trigger thrombosis due to higher shear zones and can stimulate thrombus growth due to increased platelet collisions. Larger platelet size, as is seen in diabetic patients, would enhance the thrombosis and occlusion.

The effects of the arteriole tortuosity were studied for several levels of tortuosity. When the same pressure drop was used for different tortuous channels, the flow rate increased for vessels with lower TI. This resulted in a higher shear stress in vessels with a lower TI, hence an increment in the number of activated platelets in corresponding cases. For cases where the initial flow rate was the same, the shear stress increased as the tortuosity index increased. Additionally, the zone where shear stress values rise above the critical shear stress threshold expands. All of these factors raise the probability of platelet activation; therefore, the initiation of thrombi is faster for more tortuous channels. However, once the thrombus began to form, the effect of the tortuosity of the channel reduced, and the main cause for the activation of platelet and evolution of thrombi is the adhesion between non-activated platelets and the thrombus they collided with. Combined, the simulation results are consistent with previous ones shown in the literature and *in vitro* experiments [9]. Since tortuous arterioles are widely observed in various tissues and organ in humans, especially in human hearts and brains [1–3], these results have wide applications in understanding the mechanism of thrombus formation in human organs.

Large platelets that are observed in diabetes likely produce more thrombotic factors, stimulate thrombopoiesis, are more sensitive to platelet agonists, and are associated with vascular damage [33,54,55]. Our results demonstrate that a larger platelet size increases the formation and development of the thrombus. Large platelet size increases the thrombus initiation by increased platelet activation and increased collision. Larger platelets also increase the size of the thrombus. Eventually, the thrombus blocks the flow and reduces the flow rate dramatically. Hence, in addition to biochemical factors associated with large platelets, as well as hypercoagulability in diabetes [56], our results suggest that the enhanced physical interactions due to large platelet size could also contribute to the high prevalence of thrombus formation in type II diabetic patients.

The assumption of 2D flow was made for computational efficiency and was in accord with most previous computational studies with large numbers of individual platelets in thrombus formation [19,20,28]. Additionally, our previous work showed that aggregates of cells in 2D flows were quantitatively similar in size and shape to those in three-dimensional (3D) flows [57]. Hence, we expected that platelet aggregates and thrombi in 2D flow simulations would be similar to 3D.

For computational feasibility, our model did not resolve the increase in adhesion propensity that occurs from the time of initial attachment to platelet spreading and thrombus stabilization [58,59]. To our knowledge, no computational studies that include activation of individual platelets in thrombosis model the increase in adhesion due to platelet spreading, likely due to computational cost. However, a computational study by Flamm et al. [26] has utilized a time-dependent model of platelet adhesion/aggregation rate that was a function of four different platelet agonists that were tracked throughout the simulations. Similarly in our future work, we will include a model of the time-dependent effects of platelet spreading on adhesion propensity. Because we assumed activated platelets were subjected to the higher adhesive strength present after platelet spreading and stabilization, our model may have produced thrombi that formed more quickly, though the *relative* effects of tortuosity (or platelet size) involved in thrombosis were expected to be similar.

Red blood cells were excluded from simulations because they are generally not present in microvascular thrombi [14]. However, red blood cells are known to push platelets toward vessel walls in straight vessels, and this effect greatly increases the concentration of platelets near walls [60,61]. Hence, we would expect that red blood cells would cause thrombosis to be initiated more quickly than in our simulations. Additionally, after the onset of thrombosis, thrombi and occlusions might form more slowly due to collisions of red blood cells that may detach platelets from thrombi, as seen in our previous work on initial thrombus formation in tortuous microvessels [61]. As well, another computational study of thrombus formation due to an injured section of a plane wall in a simple shear flow showed that although the presence of red blood cells resulted in shorter thrombi containing more platelets, the process of thrombus formation in general was not significantly affected by the presence of red blood cells [62]. Though we would expect our results to give differences between a specific simulation case with red blood cells compared to that same simulation case without red blood cells, we would expect a qualitatively similar relationship between tortuosity index and thrombotic occlusion in either the presence or absence of red blood cells. Likewise, we would expect a qualitatively similar relationship between platelet size and thrombotic occlusion. Red blood cells in whole blood also contribute to the decrease of the apparent viscosity as the diameter of a straight tube decreases, for diameters less than 300 μm (known as the *Fåhræus-Lindqvist effect*) [63]. A computational study showed that in a straight tube with diameter of 20 μm and hematocrit of 20% (as may be expected in a microvessel), viscosity was reduced, and the maximum velocity was about 20% lower than that of Poiseuille flow, while the velocity profile near the wall was the same [64]. Because the effect of vessel diameter on apparent viscosity in tortuous vessels is unknown, the straight tube results [64] suggest that exclusion of red blood cells and use of the viscosity of plasma would not greatly affect our conclusions because the velocity near the wall (where thrombosis occurs) was not greatly affected in straight tubes. Further studies are needed to determine the specific effects of red blood cells on thrombotic occlusion in tortuous vessels.

Our platelet activation model did not consider the amount of time a platelet was subjected to shear stress, which has been shown to affect shear-induced platelet activation in vitro [65–67]. Other existing models of shear-induced platelet activation have utilized an activation index as a measure of shear stress history to account for magnitude and exposure time [65,66,68,69]. With some of these models, depending on selection of adjustable parameters and amount of time examined, the activation index could continually increase under physiological conditions. In some cases, the activation index could even become larger under physiological conditions compared with pathological conditions, rather than being smaller as expected. Our previous work using DEM to simulate thrombosis in tortuous arterioles [7] compared our activation model to an activation model that considered shear history [68]. For certain parameters of this *shear history model*, the two models gave quantitatively similar results for the amount of thrombus in regions of high shear stress, thrombus size, number of platelets in contact with the wall, and number of activated platelets. For other parameters of the shear history model, platelet activation and thrombus formation first occurred at the second encountered region (2^nd^ bend of the vessel) of high shear stress with the shear history model, rather than the first region with our activation model. In this case, results of our model still agreed with those of the shear history model, except that the location of thrombus formation was shifted downstream by one wavelength (bend) with the shear history model. Note that for simplicity, both of these models focus on shear-activation without considering the many other factors involved in platelet activation, such as production and convection of platelet agonists. Our future work will include experimental tests to validate and enhance our shear-induced platelet activation model.

One innovative aspect of this study is the development of a new approach combining LBM and DEM to simulate the formation and growth of thrombus. It incorporates the fluid-platelet coupling. While the effects of tortuosity and platelet size on thrombus formation are similar to previous results [7,8], which validated our results, the novelty of this study lies in the capability to account for the effect of thrombus on the fluid field. These results shed new light on the growth of thrombus and occlusion process in microvasculature.

In conclusion, we developed a new approach which combines the LBM and DEM to simulate the formation and growth of thrombus in tortuous microvessels. Our simulation results demonstrate that vessel tortuosity can trigger the formation of thrombus that could lead to occlusion, agreeing with previous experimental observation. The use of LBM allows us to compute flow fields in complex microvessels more efficiently, which is critical in fluid-platelet coupling modeling. Furthermore, the use of a four-way coupling (fluid-platelet and platelet-platelet) DEM enables us to uncover the influence of the thrombus formation and growth on the flow velocity, shear stress, and flow rate. The current approach can serve as a tool for further studies of thrombosis in other pathological conditions such as in stenotic or stented arteries and may lead to better treatment selection and therapy development.

## Figures and Tables

**Table 1:** Comparisons of LBM simulation results and exact solutions.

Δ*p* (Pa)	Flow rate (10^−8^×m^2^/s)			Wall shear stress (Pa)		
	Exact	LBM	Difference (%)	Exact	LBM	Difference (%)
5	1.68	1.64	2.38	0.199	0.191	4.02
10	3.36	3.28	2.38	0.398	0.383	3.77
15	5.03	4.93	1.39	0.597	0.576	3.52
20	6.71	6.51	2.98	0.796	0.768	3.52
30	10.06	9.88	1.80	1.194	1.152	3.52

**Table 2:** Prescribed constant pressure drop in microvessels at three tortuosity levels when the initial flow rate is the same.

TI	Δ*p* (Pa)	Flow rate (10^−8^×m^2^/s)
0.08	17.30	5.394
0.16	20.00	5.393
0.24	24.47	5.394

## References

[1] Han HC (2012). Twisted blood vessels: symptoms, etiology and biomechanical mechanisms. J Vasc Res.

[2] Hutchins GM, Miner MM, Bulkley BH (1978). Tortuosity as an Index of Age and Diameter Increase of Coronary Collateral Vessels in Patients after Acute Myocardial-Infarction. Am J Cardiol.

[3] Spangler KM, Challa VR, Moody DM, Bell MA (1994). Arteriolar tortuosity of the white matter in aging and hypertension. A microradiographic study. J Neuropathol Exp Neurol.

[4] Brown WR, Moody DM, Challa VR, Thore CR, Anstrom JA (2002). Venous collagenosis and arteriolar tortuosity in leukoaraiosis. J Neurol Sci.

[5] Sasongko MB, Wong TY, Donaghue KC, Cheung N, Jenkins AJ (2012). Retinal Arteriolar Tortuosity is Associated With Retinopathy and Early Kidney Dysfunction in Type 1 Diabetes. Am J Ophthalmol.

[6] Owen CG, Newsom RS, Rudnicka AR, Barman SA, Woodward EG (2008). Diabetes and the tortuosity of vessels of the bulbar conjunctiva. Ophthalmology.

[7] Chesnutt JK, Han HC (2013). Platelet size and density affect shear-induced thrombus formation in tortuous arterioles. Phys Biol.

[8] Chesnutt JKW, Han HC (2011). Tortuosity triggers platelet activation and thrombus formation in microvessels. J Biomech Eng.

[9] Liu Q, Mirc D, Fu BM (2008). Mechanical mechanisms of thrombosis in intact bent microvessels of rat mesentery. Journal of Biomechanics.

[10] Gando S (2010). Microvascular thrombosis and multiple organ dysfunction syndrome. Crit Care Med.

[11] Taylor FB (2001). Staging of the pathophysiologic responses of the primate microvasculature to Escherichia coli and endotoxin: examination of the elements of the compensated response and their links to the corresponding uncompensated lethal variants. Crit Care Med.

[12] Sasongko MB, Wang JJ, Donaghue KC, Cheung N, Benitez-Aguirre P (2010). Alterations in retinal microvascular geometry in young type 1 diabetes. Diabetes Care.

[13] Cheung AT, Ramanujam S, Greer DA, Kumagai LF, Aoki TT (2001). Microvascular abnormalities in the bulbar conjunctiva of patients with type 2 diabetes mellitus. Endocr Pract.

[14] Rumbaut RE, Slaff DW, Burns AR (2005). Microvascular thrombosis models in venules and arterioles in vivo. Microcirculation.

[15] Wootton DM, Ku DN (1999). Fluid mechanics of vascular systems, diseases, and thrombosis. Annu Rev Biomed Eng.

[16] Para A, Bark D, Lin A, Ku D (2011). Rapid platelet accumulation leading to thrombotic occlusion. Annals of Biomedical Engineering.

[17] Para AN, Ku DN (2013). A low-volume, single pass in-vitro system of high shear thrombosis in a stenosis. Thromb Res.

[18] Wootton DM, Markou CP, Hanson SR, Ku DN (2001). A mechanistic model of acute platelet accumulation in thrombogenic stenoses. Ann Biomed Eng.

[19] Mori D, Yano K, Tsubota K, Ishikawa T, Wada S (2008). Simulation of platelet adhesion and aggregation regulated by fibrinogen and von Willebrand factor. Thromb Haemost.

[20] Miyazaki H, Yamaguchi T (2003). Formation and destruction of primary thrombi under the influence of blood flow and von Willebrand factor analyzed by a discrete element method. Biorheology.

[21] Kamada H, Tsubota K, Nakamura M, Wada S, Ishikawa T (2010). A three-dimensional particle simulation of the formation and collapse of a primary thrombus. Int J Numer Meth Bio.

[22] Filipovic N, Kojic M, Tsuda A (2008). Modelling thrombosis using dissipative particle dynamics method. Philos T R Soc A.

[23] Xu ZL, Chen N, Shadden SC, Marsden JE, Kamocka MM (2009). Study of blood flow impact on growth of thrombi using a multiscale model. Soft Matter.

[24] Pivkin IV, Richardson PD, Karniadakis G (2006). Blood flow velocity effects and role of activation delay time on growth and form of platelet thrombi. P Natl Acad Sci USA.

[25] Fogelson AL, Guy RD (2008). Immersed-boundary-type models of intravascular platelet aggregation. Comput Method Appl M.

[26] Flamm MH, Colace TV, Chatterjee MS, Jing H, Zhou S (2012). Multiscale prediction of patient-specific platelet function under flow. Blood.

[27] Kamada H, Tsubota K, Nakamura M, Wada S, Ishikawa T (2011). Computational study on effect of stenosis on primary thrombus formation. Biorheology.

[28] Flamm MH, Sinno T, Diamond SL (2011). Simulation of aggregating particles in complex flows by the lattice kinetic Monte Carlo method. J Chem Phys.

[29] Polanczyk AMP, Stefanczyk Ludomir, Szubert Wojciech, Zbicinski Ireneusz (2015). A 3D model of thrombus formation in a stent-graft after implantation in the abdominal aorta. Journal of Biomechanics.

[30] Govindarajan V, Rakesh V, Reifman J, Mitrophanov AY (2016). Computational Study of Thrombus Formation and Clotting Factor Effects under Venous Flow Conditions. Biophys J.

[31] Zimny S, Chopard B, Malaspinas O, Lorenz E, Jain K (2013). A multiscale approach for the coupled simulation of blood flow and thrombus formation in intracranial aneurysms. Procedia Comput Sci.

[32] Karpatkin S (1978). Heterogeneity of human platelets. VI. Correlation of platelet function with platelet volume. Blood.

[33] Papanas N, Symeonidis G, Maltezos E, Mavridis G, Karavageli E (2004). Mean platelet volume in patients with type 2 diabetes mellitus. Platelets.

[34] Cambronero F, Marin F, Roldan V, Hernandez-Romero D, Valdes M (2009). Biomarkers of pathophysiology in hypertrophic cardiomyopathy: implications for clinical management and prognosis. European Heart Journal.

[35] Chu SG, Becker RC, Berger PB, Bhatt DL, Eikelboom JW (2010). Mean platelet volume as a predictor of cardiovascular risk: a systematic review and meta-analysis. J Thromb Haemost.

[36] Guvenc TS, Erer HB, Ilhan S, Zeren G, Ilhan E (2012). Comparison of mean platelet volume values among different causes of pulmonary hypertension. Cardiol J.

[37] Mhawech P, Saleem A (2000). Inherited giant platelet disorders. Classification and literature review. Am J Clin Pathol.

[38] Erdem E, Erdem D, Dilek M, Kaya C, Karatas A (2012). Red Cell Distribution Width and Mean Platelet Volume in Amyloidosis. Clin Appl Thromb Hemost.

[39] Ochs HD, Slichter SJ, Harker LA, Von Behrens WE, Clark RA (1980). The Wiskott-Aldrich syndrome: studies of lymphocytes, granulocytes, and platelets. Blood.

[40] Ouared R, Chopard B (2005). Lattice Boltzmann Simulations of Blood Flow: Non-Newtonian Rheology and Clotting Processes. J Stat Phys.

[41] Zhang JF, Johnson PC, Popel AS (2008). Red blood cell aggregation and dissociation in shear flows simulated by lattice Boltzmann method. Journal of Biomechanics.

[42] Sun C, Migliorini C, Munn LL (2003). Red blood cells initiate leukocyte rolling in postcapillary expansions: a lattice Boltzmann analysis. Biophys J.

[43] Aidun CK, Clausen JR (2010). Lattice-Boltzmann Method for Complex Flows. Annual Review of Fluid Mechanics.

[44] He X, Luo L-S (1997). Theory of the lattice Boltzmann method: From the Boltzmann equation to the lattice Boltzmann equation. Physical Review E.

[45] Feng ZG, Michaelides EE (2004). The immersed boundary-lattice Boltzmann method for solving fluid-particles interaction problems. J Comput Phys.

[46] Chesnutt JKW, Marshall JS (2009). Blood cell transport and aggregation using discrete ellipsoidal particles. Comput Fluids.

[47] Feng ZG, Mao S, Michaelides EE (2010). A Three-Dimensional Resolved Discrete Particle Method for Studying Particle-Wall Collision in a Viscous Fluid. Journal of Fluids Engineering.

[48] Wu Z, Xu Z, Kim O, Alber M (2014). Three-dimensional multi-scale model of deformable platelets adhesion to vessel wall in blood flow. Philosophical transactions Series A, Mathematical, physical, and engineering sciences.

[49] Lam WA, Chaudhuri O, Crow A, Webster KD, Li TD (2011). Mechanics and contraction dynamics of single platelets and implications for clot stiffening. Nat Mater.

[50] Litvinov RI, Bennett JS, Weisel JW, Shuman H (2005). Multi-step fibrinogen binding to the integrin (alpha)IIb(beta)3 detected using force spectroscopy. Biophys J.

[51] Reininger AJ, Heijnen HF, Schumann H, Specht HM, Schramm W (2006). Mechanism of platelet adhesion to von Willebrand factor and microparticle formation under high shear stress. Blood.

[52] Romero G, Martinez ML, Maroto J, Felez J (2013). Blood Clot Simulation Model by Using the Bond-Graph Technique. The Scientific World Journal.

[53] Tang D, Yang CN, Ku D (1999). A 3-D thin-wall model with fluid–structure interactions for blood flow in carotid arteries with symmetric and asymmetric stenoses. Computers & Structures.

[54] Sharpe PC, Trinick T (1993). Mean platelet volume in diabetes mellitus. Q J Med.

[55] Jaremo P, Milovanovic M, Lindahl TL, Richter A (2009). Elevated platelet density and enhanced platelet reactivity in stable angina pectoris complicated by diabetes mellitus type II. Thromb Res.

[56] Carr ME (2001). Diabetes mellitus: a hypercoagulable state. J Diabetes Complications.

[57] Chesnutt JKW, Marshall JS (2010). Structural Analysis of Red Blood Cell Aggregates Under Shear Flow. Annals of Biomedical Engineering.

[58] Hartwig JH, Michelson AD (2007). Chapter 4 - The platelet cytoskeleton. Platelets.

[59] Savage B, Ruggeri ZM, Michelson AD (2007). Chapter 18 - Platelet thrombus formation in flowing blood. Platelets.

[60] Aarts PA, van den Broek SA, Prins GW, Kuiken GD, Sixma JJ (1988). Blood platelets are concentrated near the wall and red blood cells, in the center in flowing blood. Arteriosclerosis.

[61] Chesnutt JK, Han HC (2013). Effect of Red Blood Cells on Platelet Activation and Thrombus Formation in Tortuous Arterioles. Frontiers Bioeng Biotech.

[62] Mori D, Yano K, Tsubota K, Ishikawa T, Wada S (2008). Computational study on effect of red blood cells on primary thrombus formation. Thromb Res.

[63] Fåhræus R, Lindqvist T (1931). THE VISCOSITY OF THE BLOOD IN NARROW CAPILLARY TUBES1931.

[64] Bagchi P (2007). Mesoscale simulation of blood flow in small vessels. Biophys J.

[65] Bluestein D, Niu L, Schoephoerster RT, Dewanjee MK (1997). Fluid mechanics of arterial stenosis: relationship to the development of mural thrombus. Annals of Biomedical Engineering.

[66] Sheriff J, Soares JS, Xenos M, Jesty J, Bluestein D (2013). Evaluation of shear-induced platelet activation models under constant and dynamic shear stress loading conditions relevant to devices. Ann Biomed Eng.

[67] Hellums JD (1994). 1993 Whitaker Lecture: biorheology in thrombosis research. Annals of Biomedical Engineering.

[68] Alemu Y, Bluestein D (2007). Flow-induced platelet activation and damage accumulation in a mechanical heart valve: numerical studies. Artif Organs.

[69] Nobili M, Sheriff J, Morbiducci U, Redaelli A, Bluestein D (2008). Platelet activation due to hemodynamic shear stresses: damage accumulation model and comparison to in vitro measurements. ASAIO J.

